# Cell Lineage-Specific Differences in Clinical Behavior of Non-Functioning Pituitary Adenomas

**DOI:** 10.1210/clinem/dgaf112

**Published:** 2025-02-21

**Authors:** Loren S van der Hoeven, Tessa N A Slagboom, Arjan Malekzadeh, Jantien Hoogmoed, Madeleine L Drent, Eleonora Aronica, Dirk Jan Stenvers, Alberto M Pereira

**Affiliations:** Amsterdam UMC, University of Amsterdam, Department of Endocrinology and Metabolism, Amsterdam Gastroenterology Endocrinology and Metabolism (AGEM), 1105 AZ, Amsterdam, the Netherlands; Amsterdam UMC, University of Amsterdam, Department of Endocrinology and Metabolism, Pituitary Center Amsterdam, 1105 AZ, Amsterdam, the Netherlands; European Reference Network on Rare Endocrine Conditions (Endo-ERN), Coordinated from Amsterdam UMC, University of Amsterdam, Department of Endocrinology and Metabolism, 1105 AZ, Amsterdam, the Netherlands; Amsterdam UMC, University of Amsterdam, Department of Endocrinology and Metabolism, Pituitary Center Amsterdam, 1105 AZ, Amsterdam, the Netherlands; Amsterdam UMC, Vrije Univerisiteit Amsterdam, Department of Endocrinology and Metabolism, Amsterdam Gastroenterology Endocrinology and Metabolism (AGEM), 1084 HV, Amsterdam, the Netherlands; Amsterdam UMC, University of Amsterdam, Medical Library, 1105 AZ, Amsterdam, the Netherlands; Amsterdam UMC, University of Amsterdam, Department of Endocrinology and Metabolism, Pituitary Center Amsterdam, 1105 AZ, Amsterdam, the Netherlands; European Reference Network on Rare Endocrine Conditions (Endo-ERN), Coordinated from Amsterdam UMC, University of Amsterdam, Department of Endocrinology and Metabolism, 1105 AZ, Amsterdam, the Netherlands; Amsterdam UMC, University of Amsterdam, Department of Neurosurgery, 1105 AZ, Amsterdam, the Netherlands; Amsterdam UMC, University of Amsterdam, Department of Endocrinology and Metabolism, Pituitary Center Amsterdam, 1105 AZ, Amsterdam, the Netherlands; European Reference Network on Rare Endocrine Conditions (Endo-ERN), Coordinated from Amsterdam UMC, University of Amsterdam, Department of Endocrinology and Metabolism, 1105 AZ, Amsterdam, the Netherlands; Amsterdam UMC, Vrije Univerisiteit Amsterdam, Department of Endocrinology and Metabolism, Amsterdam Gastroenterology Endocrinology and Metabolism (AGEM), 1084 HV, Amsterdam, the Netherlands; Amsterdam UMC, University of Amsterdam, Department of Endocrinology and Metabolism, Pituitary Center Amsterdam, 1105 AZ, Amsterdam, the Netherlands; Amsterdam UMC, University of Amsterdam, Department of (Neuro)Pathology, 1105 AZ, Amsterdam, the Netherlands; Amsterdam UMC, University of Amsterdam, Department of Endocrinology and Metabolism, Amsterdam Gastroenterology Endocrinology and Metabolism (AGEM), 1105 AZ, Amsterdam, the Netherlands; Amsterdam UMC, University of Amsterdam, Department of Endocrinology and Metabolism, Pituitary Center Amsterdam, 1105 AZ, Amsterdam, the Netherlands; European Reference Network on Rare Endocrine Conditions (Endo-ERN), Coordinated from Amsterdam UMC, University of Amsterdam, Department of Endocrinology and Metabolism, 1105 AZ, Amsterdam, the Netherlands; Amsterdam UMC, Vrije Univerisiteit Amsterdam, Department of Endocrinology and Metabolism, Amsterdam Gastroenterology Endocrinology and Metabolism (AGEM), 1084 HV, Amsterdam, the Netherlands; Amsterdam UMC, University of Amsterdam, Department of Endocrinology and Metabolism, Amsterdam Gastroenterology Endocrinology and Metabolism (AGEM), 1105 AZ, Amsterdam, the Netherlands; Amsterdam UMC, University of Amsterdam, Department of Endocrinology and Metabolism, Pituitary Center Amsterdam, 1105 AZ, Amsterdam, the Netherlands; European Reference Network on Rare Endocrine Conditions (Endo-ERN), Coordinated from Amsterdam UMC, University of Amsterdam, Department of Endocrinology and Metabolism, 1105 AZ, Amsterdam, the Netherlands

**Keywords:** pituitary adenoma, PitNET, transcription factors, SF1, TPIT, null cell adenoma

## Abstract

**Context:**

Immunohistochemistry (IHC) of cell lineage-specific transcription factors (TFs) has been added to the histopathological classification of pituitary adenomas since 2017, resulting in new histopathological subtypes of TF+/hormone−non-functioning pituitary adenomas (NFPAs) and a reduction in the prevalence of null cell adenomas (NCAs).

**Objective:**

This work aimed to evaluate associations between expression of cell lineage-specific TFs by IHC and radiological invasion and prognosis of NFPAs.

**Data sources:**

A literature search in Medline, Embase, and CENTRAL was performed from inception up to July 11, 2023.

**Study selection:**

Eligible studies were cohort studies reporting on radiological invasion, recurrence, and/or radiotherapy in patients with NFPAs who tested positive for one cell lineage-specific TF or negative for all 3. Finally, 27 out of 1985 studies were included.

**Data extraction:**

Two authors independently extracted data and critically appraised risk of bias using the Quality In Prognostic Studies (QUIPS) tool.

**Data synthesis:**

Random-effects inverse variance models were used to pool effect sizes. Prevalence rate ratios (PRRs) were calculated using the Mantel-Haenszel method. Cavernous sinus invasion was more prevalent in NCAs and TPIT+ NFPAs compared with SF1+ NFPAs (PRR 1.60; 95% CI, 1.22-2.08, *I^2^* 10%, 95% prediction interval [PrI] 1.23-2.06; *P* = .0036, and PRR 1.43; 95% CI, 1.21-1.70, *I^2^* 0%, 95% PrI 1.17-1.76; *P* = .0017, respectively), and in NCAs compared with PIT1+ (PRR 1.44; 95% CI, 1.01-2.06, *I^2^* 0%, 95% PrI 0.83-2.50; *P* = .0454). A limited number of studies precluded data syntheses of recurrence and radiotherapy.

**Conclusion:**

The use of cell lineage-specific TFs by IHC enables to detect histopathological subtypes of NFPAs with distinct clinical behavior.

Pituitary adenomas (PAs), also referred to as pituitary neuroendocrine tumors (PitNETs), are intracranial tumors derived from the adenohypophysis. Clinically apparent non-functioning pituitary adenomas (NFPAs) are rare, with a prevalence of 1 to 5:10 000. Before 2017, the pathological classification of PAs was based on hormone secretion, immunohistochemistry (IHC) of adenohypophyseal hormones, and ultrastructural characteristics (1). In 2017, the World Health Organization (WHO) revised the pathological classification of PAs, incorporating IHC of cellular lineage-specific transcription factors (TFs) (ENDO4) (2). These TFs include steroidogenic factor 1 (SF1) for gonadotroph cell differentiation, T-box family member TBX19 (TPIT) for corticotroph cell differentiation, and pituitary transcription factor 1 (PIT1) for somatotroph, lactotroph, and thyrotroph cell differentiation. Additionally, nonspecific TFs GATA binding protein 2/3 (GATA2/3) and estrogen receptor α can be used to further classify PAs into gonadotroph and thyrotroph, and gonadotroph and lactotroph PAs, respectively. The WHO 2022 pathological classification maintains the use of TFs (ENDO5) (3).

Implementation of the 2017 WHO pathological classification has led to shifts in histopathological diagnosis and to newly identified subtypes within the group of NFPAs that was previously (according to WHO 2004) classified as null cell adenoma (IHC adenohypophyseal hormone negative [H−] NFPAs). As a consequence, the definitions of gonadotroph and corticotroph NFPAs and somatomammothyrotroph adenomas have been expanded to include NFPAs with IHC positivity of the corresponding cell lineage-specific TF, despite negative IHC of the adenohypophyseal hormones (TF+/H− NFPAs). In other words, silent gonadotroph adenomas (SGAs) now also include SF1+/luteinizing hormone (LH)−/follicle-stimulating hormone (FSH)− NFPAs, and silent corticotroph adenomas (SCAs) now include TPIT+/adrenocorticotropin (ACTH)− NFPAs, and so forth. Conversely, the definition of true null cell adenomas (NCAs) has been refined to encompass only adenomas that, on IHC analysis, exhibit a negative result for all adenohypophyseal hormones and TFs (TF−/H− NFPAs). Multiple studies have shown that the use of the cell lineage-specific TFs will identify the majority of IHC H− NFPAs as SGAs or SCAs (4-6).

The question is whether identification of the newly defined histopathological subtypes will also aid clinical decision-making by identifying subtypes of NFPAs with distinct clinical behavior. It is too simplistic to assume that TF+/H− NFPAs mimic the clinical behavior of the IHC H+ subtypes within the same cell lineage, or that TF−/H− NFPAs imitate the behavior of IHC H− NFPAs. The WHO 2017 classification classified sparsely granulated somatotroph adenomas, lactotroph adenomas in men, Crooke's cell adenoma, SCAs and plurihormonal PIT1 adenomas as “high-risk pituitary adenomas” (2, 7). In the 2022 revision, the WHO refers to “more aggressive adenomas,” including Crooke cell adenomas, NCAs, SCAs, and immature PIT1 lineage adenomas (ENDO5) (7). However, evidence of differences in clinical behavior of the newly identified subtypes of NFPAs is limited due to the rarity of some pathological subtypes and the variety of definitions used to define aggressive behavior. Prior to the introduction of the WHO 2017 classification, available studies were inconclusive with regard to differences in clinical behavior of NFPAs based on the histopathological diagnosis. Some studies stated that IHC H− NFPAs might have favorable outcomes in terms of long-term recurrence compared with IHC H+ NFPAs (8), especially IHC ACTH+ NFPAs (9). A more recent systematic review and meta-analysis, published in 2018, concluded that IHC ACTH+ NFPAs did not have a higher recurrence rate (RR) compared with other NFPAs (10). Nevertheless, IHC of TFs were not taken into account in any of these studies.

Now, several years after the introduction of TFs in the histopathological classification, more data have become available on the clinical, pathological, and radiological characteristics, and clinical outcomes of cell lineage-specific NFPAs (5), also including the newly identified TF+/H− subtypes (6, 11-14). Differences in clinical behavior between histopathological subtypes of NFPAs may affect clinical management strategies, surveillance, and/or follow-up. To our knowledge, to date, no systematic review or meta-analysis has comprehensively reviewed the available literature on differences in clinical behavior by histopathological subtype of NFPAs following the introduction of TFs. Therefore, it remains unclear if exposure to 1 of the 4 cell lineage-specific TF patterns (SF1+, TPIT+, PIT1+, or negativity for all 3 TFs [NCA]) affects presentation and prognosis. In other words, the additional clinical effect of detection of the cell lineage-specific TFs by IHC (WHO 2017/2022 histopathological classification) of NFPAs remains unclear.

In this systematic review and meta-analysis, we evaluate the evidence relating the exposure to different histopathological expression patterns of cell lineage-specific TFs and anterior pituitary hormones to radiological invasion, and clinical prognosis in patients with a clinically NFPA.

## Materials and Methods

We conducted a systematic review and meta-analysis. The reporting of the review and meta-analysis has been in accordance with the Preferred Reporting Items for Systematic Reviews and Meta-Analyses (PRISMA) statement 2020 (15). The present study is registered with PROSPERO (registration No. CRD42023435409).

### Objectives

The primary aim of this systematic review and meta-analysis was to answer the question “Does exposure to different expression patterns of cell lineage-specific TFs affect radiological invasion and clinical prognosis in patients with NFPAs?” after which we have formed our primary PECO statement (Box 1) (16).

Box 1 **PECO statements**

**P (Population):** patients with non-functioning pituitary adenomas (NFPAs) who underwent surgery with pituitary tissue samples available for histopathological analysis

**O (Outcomes):** Radiological invasion, tumor recurrence, and postoperative multimodality treatment

Primary PECO statement

Comparison group 1

**E (Exposure):** patients who tested positive for 1 of the 4 different cell lineage-specific transcription factor (TF) expression patterns (SF1+, TPIT+, PIT1+ or negativity for all 3 of the TFs [NCA]) detected by immunohistochemistry (IHC)

**C (Comparators):** 1 of the other 3 expression patterns

Secondary PECO statements

Comparison group 2

**E (Exposure):** patients who tested positive for 1 of the 4 different cell lineage-specific TF expression patterns without positivity for anterior pitutary hormone(s) (SF1+/H−, TPIT+/H−, PIT1+/H− or negativity for all 3 of the TFs [NCA]) detected by IHC

**C (Comparators):** 1 of the other 3 expression patterns

Comparison group 3

**E (Exposure):** patients who tested positive for cell lineage-specific anterior pituitary hormone(s) detected by IHC (SF1+/H+; TPIT+/H+; PIT1+/H+)

**C (Comparators):** patients who tested negative for cell lineage-specific anterior pituitary hormone(s) within the same cell lineage detected by IHC (SF1+/H−; TPIT+/H−; PIT1+/H−)

We postulated that the histopathological subset of IHC H− NFPAs is, in fact, more heterogeneous than was previously assumed. Therefore, we were interested in this specific subgroup of NFPAs. This resulted in our secondary aim to answer 2 additional questions. First, “Does exposure to different cell lineage-specific TF expression patterns (SF1+/H−, TPIT+/H−, PIT1+/H−, or negativity for all 3 of the TFs [NCA]) affect radiological presentation and clinical prognosis in patients with IHC H− NFPAs?” Secondly, “Does exposure to H+ compared to H− within one cell lineage affect radiological presentation and clinical prognosis in patients with NFPAs?” Corresponding PECO questions are presented in Box 1 (comparison groups 2 and 3).

### Comparison Groups

**Figure 1.**
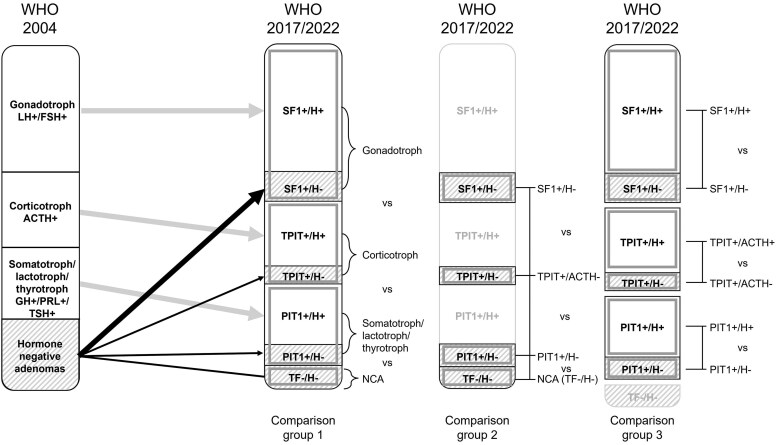
Comparison groups of histopathological subtypes of non-functioning pituitary adenomas (NFPAs). Comparison group 1: cross-comparisons between cell lineage-specific transcription factor (TF)-positive (SF1+, TPIT+, and PIT1+ NFPAs) and cell lineage-specific TF-negative (NCAs) NFPAs. Immunohistochemistry (IHC) of adenohypophyseal hormones is not taken into account. Comparison group 2: cross-comparisons between cell lineage-specific TF-positive and adenohypophyseal hormone-negative NFPAs (SF1+/H−, TPIT+/H−, and PIT1+/H−) and TF-negative NFPAs (TF–; NCAs) by IHC. Comparison group 3: cross-comparisons between cell lineage-specific TF-positive and adenohypophyseal hormone-positive, and adenohypophyseal hormone-negative NFPAs (SF1+/H− vs SF1+/H+, TPIT+/H− vs TPIT+/H+, and PIT1+/H− vs PIT1+/H+) by IHC. Abbreviations: ACTH, adrenocorticotropin; FSH, follicle-stimulating hormone; GH, growth hormone; H, adenohypophyseal hormone; IHC, immunohistochemistry; LH, luteinizing hormone; NCA, null cell adenoma; NFPA, non-functioning pituitary adenoma; PIT1, pituitary transcription factor 1; PRL, prolactin; SF1, steroidogenic factor 1; TF, transcription factor; TPIT, T-box family member TBX19; TSH, thyrotropin; WHO, World Health Organization.

To fully evaluate the effect of IHC of TFs, we have constructed 3 comparison groups in accordance with our 3 PECO statements (Fig. 1). Within comparison group 1 we examined associations between 4 histopathological subtypes of NFPAs based on their expression pattern of cell lineage-specific TFs (SF1+, TPIT+, PIT1+, or negativity for all 3 of the TFs [NCA]) and radiological invasion and clinical prognosis (primary aim). Comparison groups 2 and 3 were constructed for our secondary aims. Within comparison group 2 we focused on NFPAs that were negative for IHC staining of anterior pituitary hormones (SF1+/H−, TPIT+/H−, PIT1+/H−, or negativity for all 3 of the TFs [NCA]). Within comparison group 3 we focused on the differences in the natural course between IHC H− and IHC H+ NFPAs within 1 of the 3 cell lineages.

### Eligibility Criteria

#### Comparison group 1

Eligible study types included retrospective and prospective cohort studies, case-control studies, and case series of original data. Cross-sectional studies that examined radiological invasion at the time of surgery were also eligible. The following inclusion criteria had to be met:

1. Patients with a pathologically confirmed diagnosis of NFPA as participants, without clinical or biochemical evidence of hormonal hypersecretion (mild hyperprolactinemia up to 150 μg/L was considered to be caused by the pituitary stalk effect and accepted in the absence of clinical symptoms of a prolactinoma)
2. Surgical intervention with pituitary tissue samples available for histopathological analysis
3. Positivity for any of the following cell lineage-specific TFs (SF1+, TPIT+, or PIT1+) or negativity for all 3 (NCA) by IHC
4. One or more of the following as outcomes: radiological invasion at the time of surgery, recurrence rates (RRs), and incidence rates (IRs) of multimodality treatment
5. Preponderance of adult patients (aged ≥18 years at time of diagnosis)

Studies that included 2 or more of these 4 histopathological subtypes (SF1+, TPIT+, PIT1+, or NCA) were eligible for one or more of the comparisons (NCA vs SF1+, TPIT+ vs SF1+, PIT1+ vs SF1+, NCA vs TPIT+, NCA vs PIT1+, and TPIT+ vs PIT1+). Within comparison group 1, IHC results of anterior pituitary hormones were not taken into account.

#### Comparison group 2

Eligible studies for comparison group 2 consisted of a subgroup of those eligible for comparison group 1 that provided additional results of IHC of the anterior pituitary hormones. Specifically, studies were eligible for comparison group 2 if they included patients who tested positive for one cell lineage-specific TF and negative for all anterior pituitary hormones (TF+/H−, SF1+/H−, TPIT+/H−, or PIT1+/H−), or negative for all 3 TFs (NCA) by IHC. Studies that included 2 or more of these 4 histopathological subtypes were eligible for one or more of the comparisons (NCA vs SF1+/H−, TPIT+/H− vs SF1+/H−, PIT1+/H− vs SF1+/H−, NCA vs TPIT+/H−, NCA vs PIT1+/H−, and TPIT+/H− vs PIT1+/H−).

#### Comparison group 3

Eligible studies for comparison group 3 consisted of a subgroup of those eligible for comparison group 1 that included patients who tested positive for one cell lineage-specific TF and either negative or positive for anterior pituitary hormones (SF1+/H−, SF1+/H+, TPIT+/H−, TPIT+/H+, PIT1+/H−, or PIT1+/H+) by IHC. Studies that included both histopathological subtypes within one cell lineage (TF+/H− and TF+/H+) were eligible for one or more of the comparisons (SF1+/H− vs SF1+/H+, TPIT+/H− vs TPIT+/H+, and PIT1+/H− vs PIT1+/H+).

Exclusion criteria were case reports and case series of fewer than 3 cases of NFPAs, as well as articles that were not available in English, letters, conference abstracts, randomized controlled trials, reviews, animal studies, and articles that included a mixture of patients (ie, functioning PAs and NFPAs) without reporting the outcomes for each of the histopathological subtypes separately, as well as articles in which the outcomes could not be linked to a specific expression pattern of TFs. We sought summary estimates from published studies and did not include data from unpublished studies.

### Outcome Measures

Radiological invasion was defined as cavernous sinus invasion (CSI) and/or sphenoid invasion at time of surgery. CSI was primarily defined as Knosp-Steiner grade 3 to 4 on preoperative magnetic resonance imaging (MRI) due to its strong correlation with intraoperative CSI and gross total resection (GTR) (17, 18). Where Knosp-Steiner grades were not reported, explicit mentioning of CSI by the authors of the original articles also qualified. Recurrence was defined as postoperative radiological regrowth in the case of GTR or growth of a residual tumor, with or without the need for additional treatment. Multimodality treatment was defined as the use of at least one evidence-based treatment modality other than surgical tumor resection or debulking, such as radiotherapy or chemotherapy.

### Search Strategy and Selection

A comprehensive search was performed in 3 databases: Medline (Ovid), Embase (Ovid), and CENTRAL (Cochrane library) from inception up to July 11, 2023, in collaboration with a medical information specialist (A.M.; full search strategies are given in Supplementary Appendix A (19)). The search included controlled terms and free text terms for synonyms of “pituitary neoplasms,” combined with synonyms of “Transcription factors” and “pituitary hormones.” The search was performed without restrictions for date or languages. Duplicate articles were excluded by using an in-house made deduplication tool (DedupEndNote (version 1.0.1) (20)).

Two reviewers (L.H. and T.S.) independently screened all articles resulting from the search based on title and abstract, after which full copies of relevant studies were retrieved and assessed for eligibility based on full text. Both stages of the selection procedure were performed using Rayyan (21). Disagreements were resolved through a consensus procedure with a third reviewer (D.S.).

### Data Extraction

Data from the selected studies were independently extracted by 2 authors (L.H. and T.S.). Study characteristics that were extracted for each article included the first author and year of publication, geographical location and hospital, study design, study aim, time period of inclusion, time of follow-up (M ± SD or median [interquartile range; IQR] + range), inclusion and exclusion criteria, reported histopathological subtypes (including total number, age, and sex within these subtypes), TFs and hormones stained by IHC, antibodies used for IHC, and cutoff values for IHC positivity for both TFs and hormones, as well as definitions of the outcomes used by the original articles. Data of clinical outcomes and clinical characteristics were extracted as proportions, M ± SD, or median [IQR] + range. Data on invasion, recurrence, and multimodality treatment were extracted in crude numbers and as proportions. When appropriate, and if sample size at risk and follow-up duration were known, RRs and IRs of multimodality treatment were standardized to the number of events per 100 patient-years (PYs) at risk. This was defined as the number of cases of recurrence and multimodality treatment divided by the observed PYs and multiplied by 100. To calculate the observed PYs, we multiplied follow-up time by the number of patients at risk. In case of missing or conflicting information, study authors were contacted.

### Risk of Bias Assessment

The Quality In Prognostic Studies (QUIPS) tool was identified as the most appropriate checklist for critical appraisal (22). Two authors (L.H. and T.S.) independently rated all articles using the QUIPS tool. Discrepancies were resolved by a meeting with a third reviewer (D.S.). Domain 2 (study attrition) was rated as “low bias” if the follow-up was missing due to a cross-sectional study design. The reporting of the methods used for IHC of TFs and pituitary hormones, including the cutoff values for positivity, were used to score domain 3 (prognostic factor measurement), which was scored as “high bias” if any of this information was missing. Domain 4 (outcome measurement) was rated as “moderate bias” if there was no clear definition or method of outcome measurement, and as “high bias” if both were unclear. Domain 4 was scored for all outcomes combined, as well as for the specific outcomes invasion, recurrence, and multimodality treatment.

### Synthesis of Evidence

We conducted meta-analyses to calculate the prevalence rates (PRs) of CSI at the time of surgery, RRs per 100 PYs and IRs of postoperative radiotherapy per 100 PYs for each histopathological subtype as outlined in the description of the comparison groups.

Additionally, we conducted meta-analyses based on studies that included at least 2 histopathological subtypes within 1 comparison group to estimate the prevalence rate ratios (PRRs) of CSI at time of surgery, recurrence rate ratios (RRRs) per 100 PYs and incidence rate ratios (IRRs) of multimodality treatment per 100 PY per pairwise comparison of 2 histopathological subtypes as outlined in the description of the comparison groups.

Studies that included participants based on characteristics of aggressive clinical behavior were not included in the meta-analyses to minimize the effect of selection bias on the primary outcomes.

All analyses were repeated excluding individual patient data of patients that did not undergo their first surgery or cohorts including patients that underwent a repeat surgery without providing the individual patient data ([probable] first surgery).

#### Radiological invasion

We conducted meta-analyses to calculate PRs with a 95% CI as an effect of the expression of cell lineage-specific TFs by IHC on CSI at the time of surgery for NFPAs. Given anticipated between-study heterogeneity, a random-effects inverse variance model was used to pool effect sizes. We used Knapp-Hartung-Sidik-Jonkmann small-sample adjustments to calculate the 95% CI around the pooled effect (23, 24). A fixed continuity of 0.5 was used in case a study had no outcome in one of the arms. The Mantel-Haenszel method was used to calculate the heterogeneity variance τ^2^ for the PRs and PRRs of CSI at the time of surgery (25).

Studies were included in the meta-analysis if they reported prevalence on Knosp-Steiner grade 3 to 4 on preoperative MRI or when the authors specifically mentioned the prevalence of radiological invasion to be CSI. Studies were excluded from meta-analysis if they reported on sphenoid invasion only or did not describe the site of invasion.

#### Postoperative recurrence and multimodality treatment

We conducted meta-analyses to calculate RRs and IRs of multimodality treatment per 100 PYs with a 95% CI as an effect of the expression of cell lineage-specific TFs by IHC on recurrence and postoperative radiotherapy, respectively, of NFPAs. Given anticipated between-study heterogeneity, a random-effects inverse variance model was used to pool effect sizes. We used Knapp-Hartung-Sidik-Jonkmann small-sample adjustments to calculate the 95% CI around the pooled effect (23, 24). A fixed continuity of 0.5 was used in case a study had no outcome in one of the arms. We performed the Freeman-Tukey double arcsine transformation to address variance instability (26, 27). The DerSimonian and Laird estimator was used to calculate the heterogeneity variance τ^2^ for the RRRs and IRRs of multimodality treatment (28).

Studies were included in the meta-analyses if the provided data on recurrence and postoperative radiotherapy could be converted into events per 100 PYs.

Results of meta-analyses were reported for analyses including 3 or more studies. The statistical significance level for subtype comparisons was set at *P* less than or equal to 05. The effect measures of all studies for recurrence and multimodality treatment, including the studies that were not eligible for meta-analyses, were plotted in a bubble plot.

### Heterogeneity

Statistical heterogeneity was identified by the *Q* statistic, generated from the χ^2^ test. A *P* value less than .10 was considered statistically significant. The proportion of the observed variance that reflected variance in true effects rather than sampling error was quantified through the *I^2^* statistics. Values between 0% and 30% suggest no important heterogeneity, between 30% and 60% suggest moderate heterogeneity, between 60% and 75% suggest substantial heterogeneity, and between 75% and 100% suggest considerable heterogeneity (29). Prediction intervals (PrI) of 95%, based on the τ^2^, were calculated to reflect the amount of variance in true effects (30, 31).

### Sensitivity Analyses

#### Definition of radiological invasion

Post hoc sensitivity analyses were performed to assess the effect of varying the definition of radiological invasion at the time of surgery, and therefore the inclusion criteria for the meta-analysis, on the PRs and PRRs. Two definitions of invasion were used in the sensitivity analyses: 1) an expanded definition from CSI to “invasion,” irrespective of the specific location (not limited to the cavernous sinus or CSI combined with other locations of invasion), and 2) a refined definition that included only those cases explicitly identified as Knosp 3 to 4 on preoperative MRI. We did not change any of the statistical strategies.

#### Time of surgery

Post hoc sensitivity analyses were also performed to assess the effect whether the surgery was the first surgery or a repeat surgery for each outcome. We conducted sensitivity analyses using only data from patients at the time of 1) a certain first surgery (ie, “first surgery”), 2) a presumed first surgery in cases where the exact treatment phase was uncertain without evidence of the surgery being a repeat surgery (“probable first surgery”), and 3) both the certain and presumed first surgeries (“[probable] first surgery”). Again, we did not change the statistical strategies.

### Publication Bias

Publication bias for the comparisons of subtypes was assessed by checking funnel-plot symmetry and the Egger test (conservative threshold; *P* < .10) (32).

### Quality of Evidence

The quality of evidence was rated according to the GRADE approach, using the GRADEpro Guideline Development Tool (33, 34). The possible ratings for the overall quality of evidence were “high,” “moderate,” “low,” or “very low.” We started to rate our results, which derived from observational studies only, as low-quality evidence. Ratings could then be downgraded based on 5 domains (risk of bias, inconsistency, indirectness, imprecision, and publication bias) or upgraded based on 2 domains (large magnitude of effect and plausible residual confounding).

All statistical analyses were performed with R studio version 4.2.1. using the packages Meta, Metafor, and Forestplot.

## Results

### Study Selection

**Figure 2.**
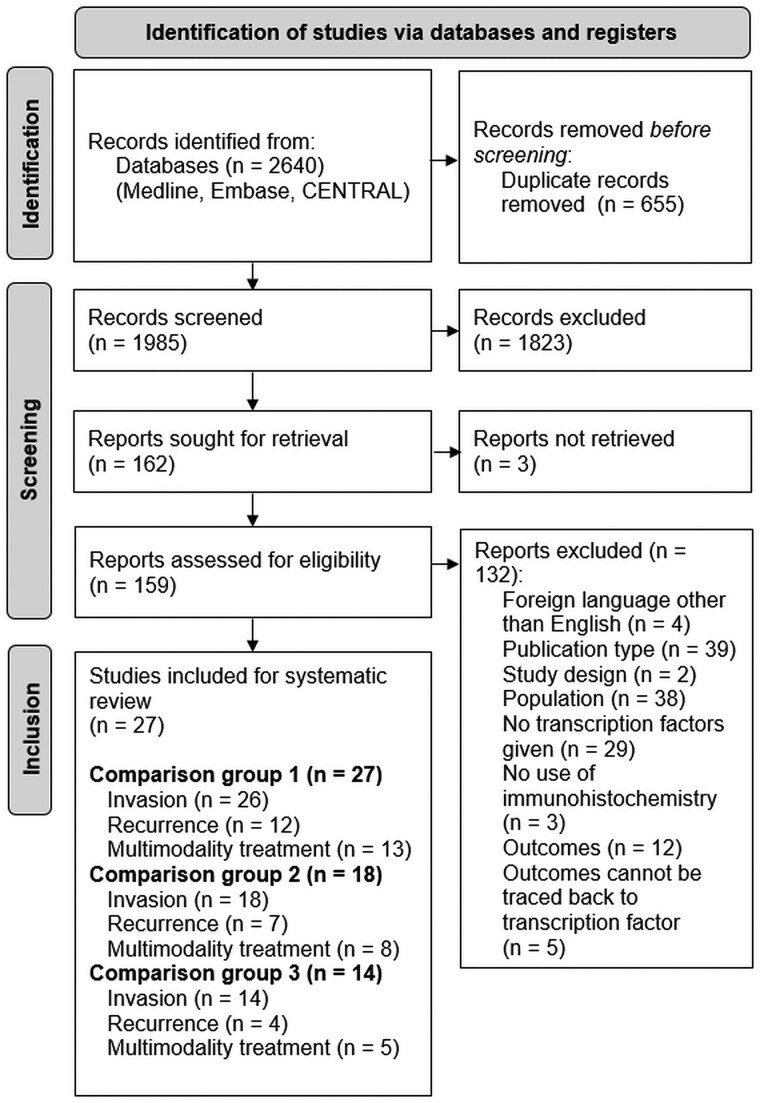
Flow diagram of included studies.

The literature search identified 1985 unique articles. After screening and assessment of eligibility, 27 articles were included in the systematic review (Fig. 2). These studies provided data on a total of 2327 patients with NFPAs (1435 SF1+, 466 TPIT+, 138 PIT1+ NFPAs, 288 NCAs). Of these, 27 studies reported data for comparison group 1, 18 provided data for comparison group 2, and 14 described outcomes for comparison group 3 (Tables 1-3, Supplementary Table S1 (19)).

**Table 1.** Summary of findings for the outcome “invasion” for comparison groups 1, 2, and 3

Study	First or repeat surgeries	Prior treatment (n/N (%))	Invasion		
			Outcome category	Population	No. of patients with (cavernous sinus) invasion (n/N (%))
**Studies included in meta-analysis of PRRs of CSI**					
Almeida 2019 (35)	Probable first surgeries		CSI	NCA	15/31 (48)
				SF1+	5/38 (13)
				SF1+/H−	5/38 (13)
Asmaro 2023 (36)	First surgeries		CSI	NCA	2/5 (40)
				SF1+	7/62 (11)
Chatrath 2022*^a,b^* (6)	First + repeat surgeries (subset analysis of first surgeries only)	Prior TSR—NCA: 8/23 (36), SF1+: 30/141 (21), TPIT+: 6/48 (13) Prior craniotomy—NCA: 0/23 (0), SF1+: 4/141 (3), TPIT+: 1/48 (2)	CSI	NCA	8/23 (35)
				SF1+	48/141 (34)
				TPIT+	18/48 (37)
Hong 2021*^c^* (37)	No indication given of prior treatment/surgery		CSI	NCA	47/111 (42)
				SF1+	26/91 (29)
				TPIT+	12/23 (52)
				PIT1+	8/29 (28)
Inoshita 2023 (38)	First + repeat surgeries	Prior surgery—NCA: 1/2 (50), SF1+: 1/2 (50), TPIT+: 0/2 (0)	CSI	NCA	1/2 (50)
				SF1+	1/2 (50)
				TPIT+	0/2 (0)
Jiang 2021 (12)	First + repeat surgeries	Prior surgery—SF1+: 21/198 (11%), TPIT+: 18/112 (16%)	CSI	SF1+	45/198 (23)
				TPIT+	43/112 (38)
				TPIT+/H−	9/33 (27)
				TPIT+/H+	34/79 (43)
Neou 2020 (39)	First + repeat surgeries	Prior surgery—NCA: 2/8 (25), SF1+: 1/28 (4), TPIT+: 2/8 (25), PIT1+: 2/8 (25%) Preoperative radiotherapy: 1/8 PIT1+ Medical therapy before surgery—NCA: 0/8 (0), SF1+: 2/28 (7), TPIT+: 1/8 (13), PIT1+: 1/8 (25)	CSI	NCA	7/8 (88)
				SF1+	13/28 (46)
				TPIT+	4/8 (50)
				PIT1+	6/8 (75)
				PIT1+/TSH+	2/3 (67)
				PIT1+/PH	4/5 (80)
Nishioka 2015*^d^* (4)	First + repeat surgeries		CSI	TPIT+	11/32 (34)
				TPIT+/H−	11/32 (34)
				PIT1+	0/2 (0)
				PIT1+/H−	0/2 (0)
Øystese 2022 (40)	First surgeries		CSI	NCA	1/4 (25)
				SF1+	20/65 (31)
				TPIT+	5/10 (50)
				PIT1+	0/4 (0)
Torregrosa-Quesada 2019 (41)	First surgeries		CSI	NCA	1/2 (50)
				SF1+	12/22 (55)
				SF1+/H+	9/15 (60)
				SF1+/H−	3/6 (50)
				TPIT+	3/5 (60)
				TPIT+/H+	3/5 (60)
				PIT1+	1/3 (33)
				PIT1+/GH+	0/1 (0)
				PIT1+/TSH+	0/1 (0)
Zhang 2023*^e^* (42)	First + repeat surgeries	Relapse status ‘relapsed’ (reported only for cohort 1)—NCA: 1/1 (100), SF1+: 1/2 (50), TPIT+: 2/3 (67), PIT1+: 1/1 (100)	CSI	NCA	12/24 (50)
				SF1+	97/312 (31)
				TPIT+	51/115 (44)
				PIT1+	6/27 (22)
**Studies not included in meta-analysis of PRRs of CSI*^f^***					
Aydin 2019*^g^* (43)	No indication given of prior treatment/surgery		CSI	PIT1+	4/9 (44)
				PIT1+/PH	4/9 (44)
Bai 2021*^g,h^* (44)	First surgeries		CSI	SF1+	8/30 (27)
Chinezu 2017*^g,h,i^* (45)	No indication given of prior treatment/surgery (“referred directly to neurosurgeon for symptoms of mass effect”)		Invasion	PIT1+	9/21 (43)
				PIT1+/GH+	1/5 (20)
				PIT1+/PH	8/16 (50)
Haddad 2020*^g^* (11)	First + repeat surgeries	Prior surgery: 29/149 (20) Preoperative radiation: 6/149 (4)	CSI	SF1+	55/149 (37)
Hickman 2021*^g,,j^* (46)	First + repeat surgeries (separate data for first surgeries only available)		CSI	SF1+	19/46 (41)
Langlois 2018*^g,i^* (47)	No indication given of prior treatment/surgery		Invasion	SF1+	29/70 (41)
Lee 2017*^g^* (48)	First surgeries		CSI	PIT1+	3/4 (75)
				PIT1+/H−	3/4 (75)
Lenders 2021*^h^* (5)	First surgeries		CSI	SF1+	5/5 (100)
				SF1+/H+	5/5 (100)
				TPIT+	1/1 (100)
				TPIT+/H−	1/1 (100)
Liu 2022*^h^* (49)	First + repeat surgeries		CSI	NCA	5/10 (50)
				SF1+	5/11 (45)
Mete 2016*^g^* (50)	First surgeries		CSI	PIT1+	5/17 (29)
				PIT1+/H−	2/5 (40)
				PIT1+/GH+	0/1 (0)
				PIT1+/PRL+	0/1 (0)
				PIT1+/PH	3/10 (30)
Micko 2019*^g,h^* (51)	First + repeat surgeries	Unknown	Other: Prevalence in Knosp 3A/3B/4 population	NCA	35/83 (42)
Micko 2020*^g^* (52)	First + repeat surgeries	Prior surgery: 4/51 (8)	CSI	SF1+	22/51 (43)
				SF1+/H+	22/51 (43)
Silva-Ortega 2021*^i^* (53)	First surgeries		Invasion	NCA	4/6 (67)
				SF1+	39/70 (56)
				PIT1+	3/3 (100)
				PIT1+/H−	3/3 (100)
Wang 2009*^g^* (54)	First surgeries	Prior therapy: 1/8 (13)	CSI	PIT1+	1/8 (13)
				PIT1+/TSH+	1/8 (13)
Zhang 2021*^g^* (55)	First + repeat surgeries	Prior surgery without radiotherapy—TPIT+: 16/105 (15) Prior surgery with radiotherapy—TPIT+: 4/105 (4)	CSI	TPIT+	76/105 (72)
				TPIT+/H+	26/39 (67)
				TPIT+/H−	50/66 (76)

Number of patients with CSI per histopathological subtype per included study sorted by eligibility for meta-analysis of PRRs. Comparison group 1: cross-comparisons between cell lineage-specific transcription factor-positive (SF1+, TPIT+, and PIT1+) NFPAs and cell lineage-specific transcription factor-negative (NCAs) NFPAs. IHC of adenohypophyseal hormones is not taken into account. Comparison group 2: cross-comparisons between cell lineage-specific transcription factor-positive and adenohypophyseal hormone-negative NFPAs (SF1+/H−, TPIT+/H−, and PIT1+/H−) and transcription factor-negative NFPAs (NCAs) by IHC. Comparison group 3: cross-comparisons between cell lineage-specific transcription factor-positive and adenohypophyseal hormone-positive, and adenohypophyseal hormone-negative NFPAs (SF1+/H− vs SF1+/H, TPIT+/H− vs TPIT+/H+, and PIT1+/H− vs PIT1+/H+) by IHC.

Abbreviations: CSI, cavernous sinus invasion; GH, growth hormone; H, adenohypophyseal hormone; IHC, immunohistochemistry; NCA, null cell adenoma; NFPA, non-functioning pituitary adenoma; PH, plurihormonal; PIT1, pituitary transcription factor 1; PRL, prolactin; PRR, prevalence rate ratio; SF1, steroidogenic factor 1; TPIT, T-box family member TBX19; TSH, thyrotropin; TSR, transsphenoidal resection.

^a^Values were estimated based on the percentage given in the individual study.

^b^Values for subset of first surgeries: NCA: 4/14 (27%), SF1+: 31/110 (28%), TPIT+: 15/41 (37%).

^c^Values were not specified in the individual study and therefore estimated based on Fig. 4 of original article.

^d^CSI defined as Knosp 4.

^e^Cohorts 1 and 2 combined.

^f^Studies excluded from meta-analysis of PRR of CSI solely based on the availability of only one pathological subtype were included in the meta-analysis of pooled PR of CSI per histopathological subtype.

^g^Excluded based on only one available pathological subtype.

^h^Excluded based on inclusion criteria of the original articles.

^i^Excluded based on no specified location of invasion.

^j^Values for subset of first surgeries: 15 of 38 (39%).

**Table 2.** Summary of findings for the outcome “recurrence” for comparison groups 1, 2, and 3

Study	First or repeat surgeries	Prior treatment Reported as n/N (%)	Follow-up, y	Population	Recurrence (n/N (%))*^a^*	RR per 100 PYs (95% CI)	Other relevant findings Reported as n/N (%), mean ± SD, or median (IQR) or else as indicated	
**Studies eligible for meta-analysis of RRRs per 100 PYs**								
Almeida 2019 (35)	Probable first surgeries		5.0 ± 5.2	NCA	5/29 (17)	6.21 (2.71-11.03)	Tumor progression-free survival at mean cohort follow-up of 5 y	0.70
			5.0 ± 5.2	SF1+	0/34 (0)	0 (0.00-1.01)		1.00
			5.0 ± 5.2	SF1+/H−	0/34 (0)	0 (0.00-1.01)		1.00
								(*P* = .011)
Jiang 2021 (12)	First + repeat surgeries	Prior surgery—SF1+: 21/198 (11%), TPIT+: 18/112 (16%), TPIT+/H+: 2/33 (6%), TPIT+/H−: 16/79 (20%)	1.2 ± 0.39 (range, 0.5-1.88)	SF1+	13/198 (7)	5.47 (2.85-8.91)		
			1.2 ± 0.38 (range, 0.5-1.8)	TPIT+	11/112 (10)	8.18 (3.96-13.84)		
			1.2 ± 0.38 (range, 0.5-1.8)	TPIT+/H+	10/79 (13)	10.55 (4.87-18.26)		
			1.2 ± 0.38 (range, 0.5-1.8)	TPIT+/H−	1/33 (3)	2.53 (0.00-10.85)		
Zhang 2023 (42)	First and repeat surgeries	Relapse status—relapsed (reported only for cohort 1): NCA: 1/1 (100), SF1+: 1/2 (50), TPIT+: 2/3 (67), PIT1+: 1/1 (100)	7.2 (6.5-8.0)	NCA	0/15 (0)	0 (0.00-1.59)	Progression-free survival, y (cohort 2)	6.90 ± 1.61
			7.2 (6.3-8.1)	SF1+	11/242 (5)	0.63 (0.31-1.07)		7.09 ± 1.20
			7.2 (6.3-8.0)	TPIT+	9/83 (11)	1.51 (0.66-2.68)		6.74 ± 1.82
			6.9 (6.3-7.2)	PIT1+	5/20 (25)	3.62 (1.01-7.64)		5.77 ± 2.48
**Studies not eligible for meta-analysis of RRRs per 100 PYs**								
Chatrath 2022*^b^* (6)	First + repeat surgeries (subset analysis of first surgeries only)	Prior TSR—NCA: 8/23 (36), SF1+: 30/141 (21), TPIT+: 6/48 (13) Prior craniotomy—NCA: 0/23 (0), SF1+: 4/141 (3), TPIT+: 1/48 (2)	8.6 ± 0.5 y (reanalyzed: 8.3 ± 0.5)	NCA		NA	Progression and recurrence HRs	Discrepancies in data in tables and figures of original article
			8.9 ± 0.3 y (reanalyzed: 9.0 ± 0.3)	SF1+				
			8.5 ± 0.4 y (reanalyzed: 8.6 ± 0.4)	TPIT+				
Haddad 2020*^c^* (11)	First + repeat surgeries	Prior surgery: 29/149 (20) Preoperative radiation: 6/149 (4)	2.4 (0-7)	SF1+	4/149 (3)	1.12 (0.24-2.55)	Time to recurrence, mo	13.5 (6-25)
Hickman 2021*^c^* (46)	First + repeat surgeries (separate data available only for first surgeries)		6 [3.5] (1.7-25)	SF1+	7/18 (39)	6.48 (2.41-12.33)	Time to recurrence, y	4.5 [IQR 5.6]
Langlois 2018*^c^* (47)	Probable first surgeries		2.7 ± 2.8	SF1+	7/70 (10)	3.70 (1.38-7.05)	Time to recurrence, mo	61 ± 85
Lee 2017*^c^* (48)	First surgeries		NR	PIT1+	3/4 (75)	NA	Time to recurrence, mo	18 (18-108)
			NR	PIT1+/H−	3/4 (75)			
Liu 2022*^d^* (49)	First + repeat surgeries		1.66 (range, 1.16-1.67)	NCA (refractory)		NA	Progression Progression after GTR or after near total resection followed by radiotherapy	12/12 (100)
			1.66 (range, 1.16-1.67)	SF1+ (refractory)		NA		4/4 (100)
			2.58 (range, 2.00-2.38)	NCA (nonrefractory)		NA		0/10 (0)
			2.58 (range, 2.00-2.38)	SF1+ (nonrefractory)		NA		0/11 (0)
Sood 2023*^c^* (56)	Probable first surgeries		NR	NCA		NA	Disease-free survival, mo	7.0 ± 1
Wang 2009*^c^* (54)	Probable first surgeries	Prior therapy: 1/8 (13)	Range, 4-20	PIT1+	1/8 (13)	NA		
			Range, 4-20	PIT1+/TSH+				
Zhang 2021*^c^* (55)	First + repeat surgeries	Prior surgery without radiotherapy—TPIT+: 16/105 (15) Prior surgery with radiotherapy—TPIT+: 4/105 (4)	1.4 ± 0.6 (range, 0.3-2.4)	TPIT+	2/46 (4)	3.11 (0.05-9.36)		

Number of patients with recurrence and calculated RRs per 100 PYs with their 95% CI per histopathological subtype per included study sorted by eligibility for meta-analysis of RRRs. Comparison group 1: cross-comparisons between cell lineage-specific transcription factor-positive (SF1+, TPIT+, and PIT1+) NFPAs and cell lineage-specific transcription factor-negative (NCAs) NFPAs. IHC of adenohypophyseal hormones is not taken into account. Comparison group 2: cross-comparisons between cell lineage-specific transcription factor-positive and adenohypophyseal hormone-negative NFPAs (SF1+/H−, TPIT+/H−, and PIT1+/H−) and transcription factor-negative NFPAs (NCAs) by IHC. Comparison group 3: cross-comparisons between cell lineage-specific transcription factor-positive and adenohypophyseal hormone-positive, and adenohypophyseal hormone-negative NFPAs (SF1+/H− vs SF1+/H, TPIT+/H− vs TPIT+/H+, and PIT1+/H− vs PIT1+/H+) by IHC.

Abbreviations: GTR, gross total resection; H, adenohypophyseal hormone; HR, hazard ratio; IHC, immunohistochemistry; IQR, interquartile range; NA, not applicable or available; NCA, null cell adenoma; NFPA, non-functioning pituitary adenoma; NR, not reported; PIT1, pituitary transcription factor 1; PY, patient-year; RR, recurrence rate; RRR, recurrence rate ratio; SF1, steroidogenic factor 1; TPIT, T-box family member TBX19; TSH, thyrotropin; TSR, transsphenoidal resection.

^a^Number of patients with recurrence/total number of patients with data on recurrence.

^b^Excluded based on discrepancies between outcomes in tables and figures in original article.

^c^Excluded based on only one available pathological subtype.

^d^Excluded based on inclusion criteria of the original article.

**Table 3.** Summary of findings for the outcome “multimodality treatment” for comparison groups 1, 2, and 3

Study	First or repeat surgeries	Follow-up, y	Prior surgery n/N (%)	Prior radiation n/N (%)	Subsequent surgery n/N (%)	Subsequent radiation n/N (%)*^a^*	Population	IR per 100 PYs—radiation after surgery (95% CI)
**Studies eligible for meta-analysis of IRRs per 100 PYs of postoperative radiotherapy**								
Almeida 2019 (35)	First surgeries	5.0 ± 5.2			4/31 (13)	5/31 (16)	NCA	3.23 (0.90-6.80)
		5.0 ± 5.2			3/38 (8)	2/38 (5)	SF1+	1.05 (0.02-3.17)
		5.0 ± 5.2			3/38 (8)	2/38 (5)	SF1+/H−	1.05 (0.02-3.17)
Jiang 2021 (12)	First and repeat surgeries	1.2 ± 0.39	21/198 (11)		5/198 (3)	8/198 (4)	SF1+	3.37 (1.37-6.17)
		1.2 ± 0.38	18/112 (16)		2/112 (2)	9/112 (8)	TPIT+	6.70 (2.92-11.90)
		1.2 ± 0.38	16/79 (20)		NR	NR	TPIT+/H+	NA
		1.2 ± 0.38	2/33 (6)		NR	NR	TPIT+/H−	NA
Zhang 2023*^b^* (42)	First and repeat surgeries	7.2 (6.5-8.0)				1/12 (8)	NCA	1.16 (0.00-4.97)
		7.2 (6.3-8.1)				25/217 (12)	SF1+	1.60 (1.03-2.29)
		7.2 (6.3-8.0)				17/70 (24)	TPIT+	3.37 (1.94-5.19)
		6.9 (6.3-7.2)				1/13 (8)	PIT1+	1.11 (0.00-4.79)
**Studies not eligible for meta-analysis of IRRs per 100 PYs of postoperative radiotherapy**								
Chatrath 2022*^c,d^* (6)	First and repeat surgeries Reanalyzed subset: first surgeries only	8.6 ± 0.5 y (reanalyzed: 8.3 ± 0.5)8.9 ± 0.3 y (reanalyzed: 9.0 ± 0.3)8.5 ± 0.4 y (reanalyzed: 8.6 ± 0.4)	9/23 (39)31/141 (22)7/48 (15)			17% (reanalyzed:14%) 18% (reanalyzed:19%) 6% (reanalyzed:4%)	NCASF1+ TPIT+	NA
Haddad 2020*^e^* (11)	First and repeat surgeries	2.4 (0-7)	29/149 (20)	6/149 (4)	7/149 (5)	14/149 (9)	SF1+	3.91 (2.10-6.27)
Langlois 2018*^e^* (47)	First surgeries	2.7 ± 2.8	0/70 (0)	0/70 (0)		2/70 (3)	SF1+	1.06 (0.02-3.19)
Lenders 2021*^f^* (5)	First surgeries	3.25 (2-5.75)	0/5 (0)		5/5 (100)	3/5 (60)	SF1+	NA
		3.25 (2-5.75)	0/5 (0)		5/5 (100)	3/5 (60)	SF1+/H+	
		3.25 (2-5.75)	0/1 (0)		1/1 (100)	1/1 (100)	TPIT+	
		3.25 (2-5.75)	0/1 (0)		1/1 (100)	1/1 (100)	TPIT+/H−	
Mete 2016*^e^* (50)	First surgeries	3.5 (IQR 4.5)			3/17 (18)	1/17 (6)	PIT1+	1.68 (0.00-7.22)
		3.5 (IQR 4.5)			0/5 (0)	1/5 (20)	PIT1+/H−	5.71 (0.00-24.55)
		3.5 (IQR 4.5)			0/1 (0)	0/1 (0)	PIT1+/GH+	0
		3.5 (IQR 4.5)			0/1 (0)	0/1 (0)	PIT1+/PRL+	0
		3.5 (IQR 4.5)			2/10 (20)	0/10 (0)	PIT1+/PH	0
Micko 2020*^e^* (52)	First and repeat surgeries	Mean 3.8 (range, 0.5-9)				2/51 (4)	SF1+	1.03 (0.02-3.11)
						2/51 (4)	SF1+/H+	1.03 (0.02-3.11)
Neou 2020*^g^* (39)	First and repeat surgeries	4.2 (3.1)	2/8 (25)	0/8 (0)			NCA	NA
		1.5 (3.33)	1/28 (4)	0/28 (0)			SF1+	
		2.5 (3)	2/8 (25)	0/8 (0)			TPIT+	
		2.5 (4.4)	2/8 (25)	1/8 (13)			PIT1+	
		3.1 (1.4-9)	1/3 (33)	0/3 (0)			PIT1+/TSH+	
		2 (4.6)	1/5 (20)	1/5 (20)			PIT1+/PH	
Zhang 2021*^e^* (55)	First and repeat surgeries	1.4 ± 0.6 (range, 0.3-2.4)	16/105 (15)	4/105 (3)		2/46 (4)	TPIT+	3.11 (0.05-9.36)
Øystese 2022*^h^* (40)	First surgeries	9.7 (8.3-12.1) 10.5 (8.3-13.2) 9.4 (8.2-14.7) 11.7 (9.8-14)	**Other outcomes**		1/4 (25) 35/100 (35) 6/17 (35) 2/8 (25) 0/4 (0) 6/100 (6) 3/17 (18) 1/8 (13)		NCA SF1+ TPIT+ PIT1+ NCA SF1+ TPIT+ PIT1+	2.58 (0.00-11.07) 3.33 (2.31-4.54) 3.75 (1.24-7.48) 2.14 (0.03-6.44) 0 (0.00-42.97) 6 (1.98-11.95) 17.65 (2.15-44.75) 12.5 (0.00-53.71)
			Reintervention (>12 mo after first surgery, both radiotherapy and surgery) Postoperative treatment (within 12 mo after first surgery)					
Liu 2022*^f,h^* (49)	First and repeat surgeries		**Treatment before and/or after surgery**					
		1.66 (range, 1.16-1.67) 1.66 (range, 1.16-1.67)	Multiple surgeries Any radiation treatment Stereotactic radiosurgery External beam radiotherapy		4/12 (33) 3/4 (75) 12/12 (100) 4/4 (100) 4/12 (33) 3/4 (75) 8/12 (67) 1/4 (25)		NCA (refractory) SF1+ (refractory)	NA

Number of patients with postoperative radiotherapy and calculated IRs and their 95% CI of postoperative radiotherapy per 100 PYs per histopathological subtype per included study sorted by eligibility for meta-analysis of IRRs. Comparison group 1: cross-comparisons between cell lineage-specific transcription factor-positive (SF1+, TPIT+, and PIT1+) NFPAs and cell lineage-specific transcription factor-negative (NCAs) NFPAs. IHC of adenohypophyseal hormones is not taken into account. Comparison group 2: cross-comparisons between cell lineage-specific transcription factor-positive and adenohypophyseal hormone-negative NFPAs (SF1+/H−, TPIT+/H−, and PIT1+/H−) and transcription factor-negative NFPAs (NCAs) by IHC. Comparison group 3: cross-comparisons between cell lineage-specific transcription factor-positive and adenohypophyseal hormone-positive, and adenohypophyseal hormone-negative NFPAs (SF1+/H− vs SF1+/H, TPIT+/H− vs TPIT+/H+, and PIT1+/H− vs PIT1+/H+) by IHC.

Abbreviations: GH, growth hormone; H, adenohypophyseal hormone; IHC, immunohistochemistry; IR, incidence rate; IRR, incidence rate ratio; NA, not applicable or available; NCA, null cell adenoma; NFPA, non-functioning pituitary adenoma; NR, not reported; PIT1, pituitary transcription factor 1; PRL, prolactin; PY, patient-year; SF1, steroidogenic factor 1; TPIT, T-box family member TBX19; TSH, thyrotropin.

^a^Number of patients with postoperative radiotherapy/total number of patients with data on postoperative radiotherapy.

^b^Based on cohort 2 (Supplementary Table S4 of original article).

^c^Excluded based on selective reporting of use of postoperative Gamma Knife treatment (only for patients who underwent treatment prior to progression of residual tumor).

^d^Values were estimated based on percentage given in the individual study.

^e^Excluded based on only one available pathological subtype.

^f^Excluded based on inclusion criteria of the original article.

^g^Excluded based on reporting of preoperative radiotherapy only.

^h^Excluded based on used definition of reintervention.

Mean/median ages ranged from 40 to 68 years. The overall proportion of women included was 47.6% (range, 23.9%-96.9% in cohorts including >10 patients). Follow-up duration ranged from 1.4 years to 14.1 years. An overview of the used antibodies for IHC are presented in Supplementary Appendix B (19). Within comparison group 1, 26 articles reported on invasion, with 23 specifically on CSI, 12 articles on recurrence, and 13 articles on multimodality treatment. Within comparison group 2, 18 articles reported on invasion, of which there were 17 on CSI specifically, 7 articles on recurrence, and 8 articles on multimodality treatment. Within comparison group 3, 14 articles reported on invasion, of which there were 12 articles on CSI, 4 articles on recurrence, and 6 articles on multimodality treatment.

### Risk of Bias Analysis

Overall, risk of bias on domain 3 (prognostic factor measurement) and domain 4 (outcome measurement) were rated as “moderate risk” or “high risk” in about half of the studies. The main reasons for assigning higher risk of bias scores were the absence of cutoff values for IHC positivity, as well as a lack of definitions and methods used to establish outcome measurements. All studies scored “moderate risk” or “high risk” on domain 5 (study confounding) (Supplementary Table S2 (19)).

### Cavernous Sinus Invasion

#### Comparison group 1

Reasons for exclusion from meta-analyses and sensitivity analyses were inclusion of patients with macroadenomas only (44), symptoms of mass effect (45), clinically aggressive tumors (5), refractory NFPAs (49), and Knosp 3 to 4 on MRI (51).

The mean pooled PR of CSI at the time of surgery was highest for TPIT+ NFPAs (PR 47%; 95% CI, 37%-58%, 10 studies, 460 participants, *I^2^* 73%, 95% PrI 21-75, low quality of evidence), followed by NCAs (PR 44%; 95% CI, 37%-51%, 9 studies, 210 participants, *I^2^* 0%, 95% PrI 36%-52%, moderate quality of evidence), SF1+ (PR 32%; 95% CI, 25%-40%, 13 studies, 1205 participants, *I^2^* 69%, 95% PrI 14%-57%, low quality of evidence), and PIT1+ NFPAs (PR 32%; 95% CI, 20%-47%, 10 studies, 111 participants, *I^2^* 27%, 95% PrI 16%-55%, low quality of evidence) (Table 4).

**Table 4.** Pooled prevalence rates of cavernous sinus invasion

	Prevalence rate of CSI at time of first and/or repeat surgery						Prevalence rate of CSI at time of (probable) first surgery					
	No. of studies	n/N	Prevalence rate (95% CI)	*I^2^*,%	95% PrI	Quality of evidence (GRADE)	No. of studies	n/N	Prevalence rate (95% CI)	*I^2^*,%	95% PrI	Quality of evidence (GRADE)
**Comparison group 1**												
SF1+	13	370/1205	0.32 (0.25-0.40)	69	0.14-0.57	⊕⊕◯◯	6	90/335	0.28 (0.14-0.47)	76	0.04-0.77	⊕◯◯◯
TPIT+	10	223/460	0.47 (0.37-0.58)	73	0.21-0.75	⊕⊕◯◯	3	23/56	0.41 (0.21-0.65)	0	0.02-0.96	⊕◯◯◯
PIT1+	10	34/111	0.32 (0.20-0.47)	27	0.16-0.55	⊕⊕◯◯	6	14/54	0.33 (0.17-0.56)	8	0.16-0.56	⊕◯◯◯
NCA	9	94/210	0.44 (0.37-0.51)	0	0.36-0.52	⊕⊕⊕◯	5	23/56	0.42 (0.29-0.55)	0	0.23-0.63	⊕⊕◯◯
**Comparison group 2**												
SF1+/H−	2	NA	NA	NA	NA	NA	2	NA	NA	NA	NA	NA
TPIT+/H−	3	70/131	0.46 (0.05-0.94)	92	0.00-1.00	⊕◯◯◯	0	NA	NA	NA	NA	NA
PIT1+/H−	3	6/12	0.49 (0.09-0.90)	0	0.00-1.00	⊕◯◯◯	3	6/12	0.49 (0.09-0.90)	0	0.00-1.00	⊕◯◯◯
NCA	9	94/210	0.44 (0.37-0.51)	0	0.36-0.52	⊕⊕⊕◯	5	23/56	0.42 (0.29-0.55)	0	0.23-0.63	⊕⊕◯◯
**Comparison group 3**												
SF1+/H−	2	NA	NA	NA	NA	NA	2	NA	NA	NA	NA	NA
SF1+/H+	2	NA	NA	NA	NA	NA	1	NA	NA	NA	NA	NA
TPIT+/H−	3	70/131	0.46 (0.05-0.94)	92	0.00-1.00	⊕◯◯◯	0	NA	NA	NA	NA	NA
TPIT+/H+	3	63/123	0.55 (0.23-0.83)	66	0.00-1.00	⊕◯◯◯	1	NA	NA	NA	NA	NA
PIT1+/H−	3	6/12	0.49 (0.09-0.90)	0	0.00-1.00	⊕◯◯◯	3	6/12	0.49 (0.09-0.90)	0	0.00-1.00	⊕◯◯◯
PIT1+/H+	5	14/39	0.36 (0.12-0.71)	44	0.03-0.92	⊕◯◯◯	4	8/31	0.28 (0.11-0.56)	0	0.06-0.70	⊕◯◯◯

Pooled prevalence rates of CSI after first and/or repeat surgery and after first surgery per histopathological subtype, sorted by comparison group. Comparison group 1: cross-comparisons between cell lineage-specific transcription factor-positive (SF1+, TPIT+, and PIT1+) NFPAs and cell lineage-specific transcription factor-negative (NCAs) NFPAs. IHC of adenohypophyseal hormones is not taken into account. Comparison group 2: cross-comparisons between cell lineage-specific transcription factor positive and adenohypophyseal hormone-negative NFPAs (SF1+/H−, TPIT+/H−, and PIT1+/H−) and transcription factor-negative NFPAs (NCAs) by IHC. Comparison group 3: cross-comparisons between cell lineage-specific transcription factor-positive and adenohypophyseal hormone-positive, and adenohypophyseal hormone-negative NFPAs (SF1+/H− vs SF1+/H, TPIT+/H− vs TPIT+/H+, and PIT1+/H− vs PIT1+/H+) by IHC.

Abbreviations: CSI, cavernous sinus invasion; H, adenohypophyseal hormone; IHC, immunohistochemistry; NA, not applicable (due to insufficient studies); NCA, null cell adenoma; NFPA, non-functioning pituitary adenoma; PIT1, pituitary transcription factor 1; PrI, prediction interval; SF1, steroidogenic factor 1; TPIT, T-box family member TBX19. ⊕◯◯◯, very low quality of evidence; ⊕⊕◯◯, low quality of evidence; ⊕⊕⊕◯, moderate quality of evidence; ⊕⊕⊕⊕, high quality of evidence.

After excluding cohorts with repeat surgeries, the pooled PRs of CSI at the time of the (probable) first surgery were marginally lower or the same, with TPIT+ NFPAs (PR 41%; 95% CI, 21%-65%, 3 studies, 56 participants, *I^2^* 0%, 95% PrI 2%-96%, very low quality of evidence) and NCAs (PR 42%; 95% CI, 29%-55%, 5 studies, 56 participants, *I^2^* 0%, 95% PrI 23%-63%, very low quality of evidence) still having the highest PR compared with SF1+ (PR 28%; 95% CI, 14%-47%, 6 studies, 335 participants, *I^2^* 0%, 95% PrI 4%-77%, very low quality of evidence), and PIT1+ NFPAs (PR 33%; 95% CI, 17%-55%, 6 studies, 54 participants, *I^2^* 8%, 95% PrI 16%-56%, very low quality of evidence) (see Table 4). However, the number of studies and sample sizes decreased clearly for some of the subtypes.

**Figure 3.**
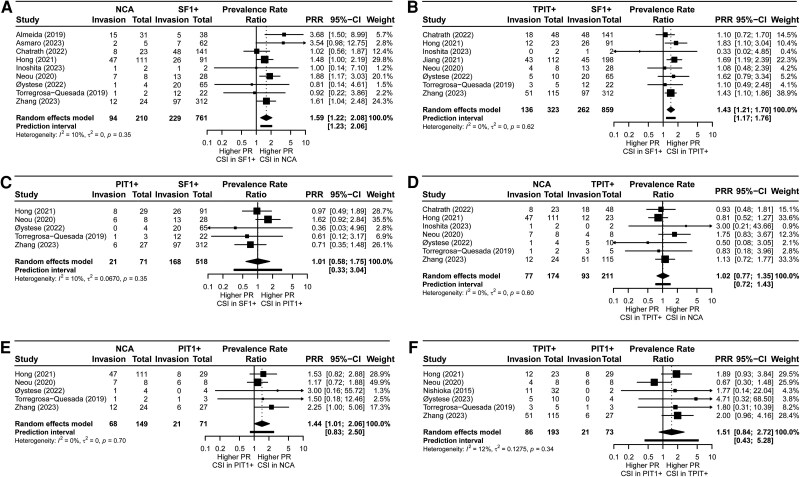
Forest plots of prevalence rate ratios (PRRs) of cavernous sinus invasion (CSI) at time of first and/or repeat surgeries for comparison group 1 of A, NCAs vs SF1+ NFPAs; B, TPIT+ vs SF1+ NFPAs; C, PIT1 + vs SF1+ NFPAs; D, NCA vs TPIT+ NFPAs; E, NCA vs PIT1+ NFPAs; and F, TPIT+ vs PIT1+ NFPAs. Comparison group 1: cross-comparisons between cell lineage-specific transcription factor-positive (SF1+, TPIT+, and PIT1+) NFPAs and cell lineage-specific transcription factor-negative (NCAs) NFPAs. Immunohistochemistry of adenohypophyseal hormones is not taken into account. Abbreviations: CSI, cavernous sinus invasion; NCA, null cell adenoma; NFPA, non-functioning pituitary adenoma; PIT1, pituitary transcription factor 1; PR, prevalence rate; PRR, prevalence rate ratio; SF1, steroidogenic factor 1; TPIT, T-box family member TBX19.

Results of the meta-analyses of the PRRs between the different subtypes were in line with the pooled PRs per subtype, with NCAs and TPIT+ NFPAs having higher prevalences of CSI at time of the first and/or repeat surgery compared with SF1+ NFPAs (PRR 1.60; 95% CI, 1.22-2.08, 9 studies, 971 participants, *I^2^* 10%, 95% PrI 1.23%-2.06%; *P* = .0036; very low quality of evidence, and PRR 1.43; 95% CI, 1.21-1.70, 8 studies, 1182 participants, *I^2^* 0%, 95% PrI 1.17%-1.76%; *P* = .0017; very low quality of evidence, respectively) and NCAs compared with PIT1+ NFPAs (PRR 1.44; 95% CI, 1.01-2.06, 5 studies, 220 participants, *I^2^* 0%, 95% PrI 0.83%-2.50%; *P* = .0454; very low quality of evidence) (Fig. 3 and Table 5).

**Table 5.** Prevalence rate ratios of cavernous sinus invasion

	PRR of CSI at time of first and/or repeat surgery							PRR of CSI at time of (probable) first surgery						
	No. of studies	n/N vs n/N	PRR (95% CI)	*I^2^*, %	95% PrI	*P*	Quality of evidence (GRADE)	No. of studies	n/N	PRR (95% CI)	*I^2^*,%	95% PrI	*P*	Quality of evidence (GRADE)
**Comparison group 1**														
**NCA vs SF1+**	9	94/210	1.60 (1.22-2.08)	10	1.23-2.06	.0036	⊕◯◯◯	5	23/56	1.73 (0.68-4.41)	41	0.24-12.32	.1813	⊕◯◯◯
		229/761							75/297					
**TPIT + vs SF1+**	8	136/323 262/859	1.43 (1.21-1.70)	0	1.17-1.76	.0017	⊕◯◯◯	3	23/56	1.33 (0.88-2.00)	0	0.12-14.35	.0955	⊕◯◯◯
									63/197					
**PIT1 + vs SF1+**	5	21/71	1.01 (0.58-1.75)	10	0.34-3.04	.9654	⊕◯◯◯	2	NA	NA	NA	NA	NA	NA
		168/518												
**NCA vs TPIT+**	7	77/174	1.02 (0.77-1.35)	0	0.72-1.43	.8812	⊕◯◯◯	3	6/20	0.74 (0.44-1.24)	0	0.01-81.69	.1291	⊕◯◯◯
		83/211							23/56					
**NCA vs PIT1+**	5	68/149	1.44 (1.01-2.06)	0	0.83-2.50	.0454	⊕◯◯◯	2	NA	NA	NA	NA	NA	NA
		21/71												
**TPIT+ vs PIT1+**	6	86/193	1.51 (0.84-2.72)	12	0.43-5.28	.1331	⊕◯◯◯	2	NA	NA	NA	NA	NA	NA
		21/73												

Pooled PPRs of CSI after first and/or repeat surgery and after first surgery between 2 histopathological subtypes. Comparison group 1: cross-comparisons between cell lineage-specific transcription factor-positive (SF1+, TPIT+, and PIT1+) NFPAs and cell lineage-specific transcription factor-negative (NCAs) NFPAs. IHC of adenohypophyseal hormones is not taken into account. Calculation of PRRs of CSI at time of first and/or repeat surgery and at time of first surgery was not possible for comparison groups 2 and 3 due to limited number of studies.

Abbreviations: CSI, cavernous sinus invasion; IHC, immunohistochemistry; NA, not applicable (due to insufficient studies); NCA, null cell adenoma; NFPA, non-functioning pituitary adenoma; PIT1, pituitary transcription factor 1; PrI, prediction interval; PRR, prevalence rate ratio; SF1, steroidogenic factor 1; TPIT, T-box family member TBX19. ⊕◯◯◯, very low quality of evidence; ⊕⊕◯◯, low quality of evidence; ⊕⊕⊕◯, moderate quality of evidence; ⊕⊕⊕⊕, high quality of evidence.

Differences in prevalence of CSI between PIT1+ compared with SF1+ NFPAs (PRR 1.01; 95% CI, 0.58-1.75, 5 studies, 589 participants, *I^2^* 10%, 95% CI, 0.34-3.04; *P* = .9654; very low quality of evidence), NCAs compared with TPIT+ NFPAs (PRR 1.02; 95% CI, 0.77-1.35, 7 studies, 385 participants, *I^2^* 0%, 95% PrI 0.72-1.43; *P* = .8812; very low quality of evidence), and TPIT+ compared with PIT1+ NFPAs could not be detected, although a trend could be seen for TPIT+ being more often invasive compared with PIT1+ NFPAs (PRR 1.51; 95% CI, 0.84-2.72, 6 studies, 266 participants, *I^2^* 12%, 95% PrI 0.43-5.28; *P* = .1331; very low quality of evidence). After excluding cohorts with patients undergoing repeat surgeries, the same direction of effects could be seen for NCAs compared SF1+ NFPAs (PRR 1.73; 95% CI, 0.68-4.41, 5 studies, 353 participants, *I^2^* 41%, PrI 0.24-12.32; *P* = .1813; very low quality of evidence), and TPIT+ vs SF1+ NFPAs (PRR 1.33; 95% CI, 0.88-2.00, 3 studies, 253 participants, *I^2^* 0%, PrI 0.12-14.35; *P* = .0955; very low quality of evidence) (see Table 5), although the numbers of studies included in the analyses were low, leading to nonsignificant CI. There was a trend toward lower frequency of CSI in NCAs compared with TPIT+ NFPAs (PRR 0.74; 95% CI, 0.44-1.24, 3 studies, 76 participants, *I^2^* 0%, 95% PrI 0.01-81.69; *P* = .1291; very low quality of evidence). All 3 studies included in this analysis showed higher prevalence rates of CSI in TPIT+ NFPAs compared with NCAs, but sample sizes were small resulting in wide CI.

#### Comparison group 2

The mean pooled PR of CSI at the time of surgery was highest in PIT1+/H− NFPAs (PR 49%; 95% CI, 9%-90%, 3 studies, 12 participants, *I^2^* 0%, 95% PrI 0%-100%, very low quality of evidence), followed by TPIT+/H− NFPAs (PR 46%; 95% CI, 5%-94%, 3 studies, 131 participants, *I^2^* 92%, 95% PrI 0%-100%, very low quality of evidence) and NCAs (PR 44%; 95% CI, 37%-51%, 9 studies, 210 participants, *I^2^* 0%, 95% PrI 36%-52%, moderate quality of evidence). PRs could not be calculated for SF1+/H− NFPAs as there were only 2 studies available that reported a wide spread of prevalences (Supplementary Fig. S1 (19)) (35, 41). Sensitivity analysis of CSI at the (probable) first surgery affected only the analysis for NCAs, showing a comparable PR (PR 42%; 95% CI, 29%-55%, 5 studies, 56 participants, *I^2^* 0%, 95% PrI 23%-63%, low quality of evidence).

Due to the limited number of studies that included 2 or more subtypes, PRRs could not be calculated by meta-analyses. A cohort study reported higher PRs of CSI in NCAs compared with SF1+/H− NFPAs (PRR 3.68; 95% CI, 1.50-8.99) (35), while another study with small sample sizes found no differences (41).

#### Comparison group 3


SF1+/H–vsSF1+/H+

Meta-analyses were not appropriate due to the limited number of studies (Supplementary Fig. S2 (19)). The one study including both SF1+/H− and SF1+/H+ NFPAs reported no statistical differences in prevalence of CSI at time of surgery (41).


TPIT+/H–vsTPIT+/H+

The mean pooled PR of CSI in TPIT+/H− and TPIT+/H+ NFPAs was 46% (95% CI, 5%-94%, 3 studies, 131 participants, *I^2^* 92%, 95% PrI 0%-100%, very low quality of evidence) and 55% (95% CI, 23%-83%, 3 studies, 123 participants, *I^2^* 66%, 95% PrI 0%-100%, very low quality of evidence), respectively. Studies including both subtypes did not detect differences in prevalence of CSI at time of surgery (12, 55).


PIT1+/H–vsPIT1+/H+

The mean pooled PR of CSI was 49% (95% CI, 9%-90%, 3 studies, 12 participants, *I^2^* 0%, 95% PrI 0%-100%, very low quality of evidence) and 36% (95% CI, 12%-71%, 5 studies, 39 participants, *I^2^* 44%, 95% PrI 3%-92%, very low quality of evidence) for PIT1+/H− and PIT1+/H+ adenomas, respectively. Meta-analysis of PRRs was inappropriate due to the limited number of studies that included both subtypes.

### Postoperative Recurrence

#### Comparison group 1

In all but 2 studies patients were treated primarily with surgery (see Table 2). Reasons for ineligibility for meta-analysis were discrepancies in data between tables and figures in the original article that could not be resolved despite correspondence with the authors (6), and based on inclusion criteria of refractory PAs matched with NFPAs (49).

**Figure 4.**
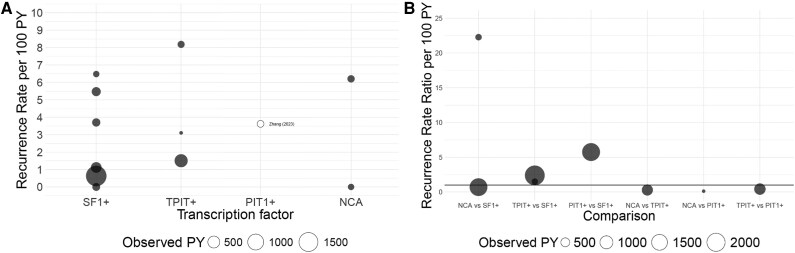
Bubble plot of A, recurrence rates per 100 patient-years (PYs) after first and/or repeat surgery by transcription factor and B, recurrence rate ratios per 100 PY by comparison for comparison group 1. Every bubble represents the recurrence rate or recurrence rate ratio calculated based on individual study data. Bubble size represents the observed PY per subtype or per comparison. Studies that are not eligible for meta-analysis are marked as a white bubble. The horizontal line on the y-axis of Fig. 4B represents a recurrence rate ratio of 1. Comparison group 1: cross-comparisons between cell lineage-specific transcription factor-positive (SF1+, TPIT+, and PIT1+) NFPAs and cell lineage-specific transcription factor-negative (NCAs) NFPAs. Immunohistochemistry of adenohypophyseal hormones is not taken into account. Abbreviations: NCA, null cell adenoma; NFPAs, non-functioning pituitary adenomas; PIT1, pituitary transcription factor 1; PY, patient-year; SF1, steroidogenic factor 1; TPIT, T-box family member TBX19.

The mean pooled recurrence rate per 100 PYs after first and/or repeat surgery was higher in TPIT+ (RR 3.64; 95% CI, 0.00-16.87, 3 studies, 796.4 observed PY, *I^2^* 84%, 95% PrI 0-16.87, very low quality of evidence) than in SF1+ NFPAs (RR 2.05; 95% CI, 0.14-5.76, 6 studies, 2804.6 observed PY, *I^2^* 88%, 95% PrI 0.14-5.76, very low quality of evidence) (Table 6 and Fig. 4). In total, 4 of 241 (2%) patients with TPIT+ adenomas were reported to have received prior surgery with radiotherapy. The recurrence rate increased marginally for SF1+ NFPAs at time of the (probable) first surgery (RR 2.39; 95% CI, 0.00-20.12, 3 studies, 467 observed PY, *I^2^* 87%, 95% PrI 0.0-308.56, very low quality of evidence), and after exclusion of cohorts in which some of the patients received preoperative radiotherapy (RR 2.34; 95% CI, 0.0-7.75, 5 studies, 2447 observed PY, *I^2^* 90%, 95% PrI 0.1-14.0, very low quality of evidence). Meta-analysis was not appropriate for PIT1+ NFPAs and NCAs.

**Table 6.** Recurrence rates per 100 patient-years

Recurrence rate per 100 PYs after first and/or repeat surgery							Recurrence rate per 100 PYs after (probable) first surgery					
	No. of studies	n/N*^a^*	Recurrence rate (95% CI)	*I^2^*, %	95% PrI	Quality of evidence (GRADE)	No. of studies	n/N*^a^*	Recurrence rate (95% CI)	*I^2^*, %	95% PrI	Quality of evidence (GRADE)
**Comparison group 1**												
SF1+	6	42/2804.6	2.05 (0.14-5.76)	88	0.00-14.00	⊕◯◯◯	3	14/467	2.39 (0.00-20.12)	87	0.00-308.56	⊕◯◯◯
Without preoperative radiotherapy	5	38/2447	2.34 (0.00-7.75)	90	0.00-22.52	⊕◯◯◯	3	14/467	2.39 (0.00-20.12)	87	0.00-308.56	⊕◯◯◯
TPIT+	3	22/796.4	3.64 (0.00-16.87)	84	0.00-245.49	⊕◯◯◯	0	NA	NA	NA	NA	NA
PIT1+	1	NA	NA	NA	NA	NA	0	NA	NA	NA	NA	NA
NCA	2	NA	NA	NA	NA	NA	1	NA	NA	NA	NA	NA

Pooled recurrence rates per histopathological subtype after first and/or repeat surgery and after first surgery. Comparison group 1: cross-comparisons between cell lineage-specific transcription factor-positive (SF1+, TPIT+, and PIT1+) NFPAs and cell lineage-specific transcription factor-negative (NCAs) NFPAs. IHC of adenohypophyseal hormones is not taken into account. Calculation of recurrence rates per 100 PYs after first and/or repeat surgery and after first surgery was not possible for comparison groups 2 and 3 due to limited number of studies.

Abbreviations: IHC, immunohistochemistry; NA, not applicable (due to insufficient studies); NCA, null cell adenoma; NFPA, non-functioning pituitary adenoma; PIT1, pituitary transcription factor 1; PrI, prediction interval; PY, patient-year; SF1, steroidogenic factor 1; TPIT, T-box family member TBX19. ⊕◯◯◯, very low quality of evidence; ⊕⊕◯◯, low quality of evidence; ⊕⊕⊕◯, moderate quality of evidence; ⊕⊕⊕⊕, high quality of evidence.

^a^The amount of patients with at least one recurrence per the amount of observed PYs.

Meta-analyses were also not appropriate for any of the subtype comparisons due to the limited number of studies reporting outcomes on multiple subtypes. A RRR of NCAs compared with SF1+ NFPAs was found in one cohort study (RRR 22.28 per 100 PY; 95% CI, 1.30-382.73; *P* = .011 by log rank test) (35), while a nonsignificant RRR of 0.70 (95% CI, 0.04-11.90) was calculated based on a second cohort study (42). Follow-up duration was 5 and 7.2 years, respectively. Calculated RRs per 100 PYs were higher in TPIT+ compared with SF1+ NFPAs after 1.2 years (RRR 1.50 per 100 PY; 95% CI, 0.67-3.34) (12) and 7.2 years (RRR 2.39 per 100 PY; 95% CI, 0.99-5.76) (42). Based on calculations from the only cohort study including all 4 histopathological subtypes, the RR was highest in PIT1+ NFPAs (RR 3.62 per 100 PY; 95% CI, 1.01-7.64), compared with the other subtypes (SF1+: RR 0.63; 95% CI, 0.31-1.07, TPIT+: RR 1.51; 95% CI, 0.66-2.68, and NCAs: RR 0.00; 95% CI, 0.00-1.59) (42) with overlapping 95% CIs.

#### Comparison group 2

Due to limited number of studies, meta-analyses of RRs per 100 PYs as well as RRRs per 100 PYs could not be performed for any subtype and/or sensitivity analysis (Supplementary Fig. S3 (19)).

#### Comparison group 3

Meta-analyses of RRs per 100 PYs as well as RRRs per 100 PYs could not be performed due to limited number of studies per subtype and comparison (Supplementary Fig. S4 (19)). A retrospective cohort study comparing TPIT+/H+ to TPIT+/H− NFPAs found no differences in recurrence after a mean follow-up of 1.2 years. The proportion of patients who had received prior surgery was higher in the TPIT+/H+ group, but this difference did not reach statistical significance (12).

### Multimodality Treatment

In the included studies, radiotherapy was the only evidence-based treatment modality used in patients besides surgery. Both preoperative and postoperative radiotherapy have been reported. Different forms of radiotherapy were reported, such as Gamma Knife radiotherapy, stereotactic radiosurgery, and external beam radiotherapy. The use of chemotherapy for PAs was reported in one study, but none of the patients with NFPAs had received chemotherapy (39).

#### Comparison group 1

**Figure 5.**
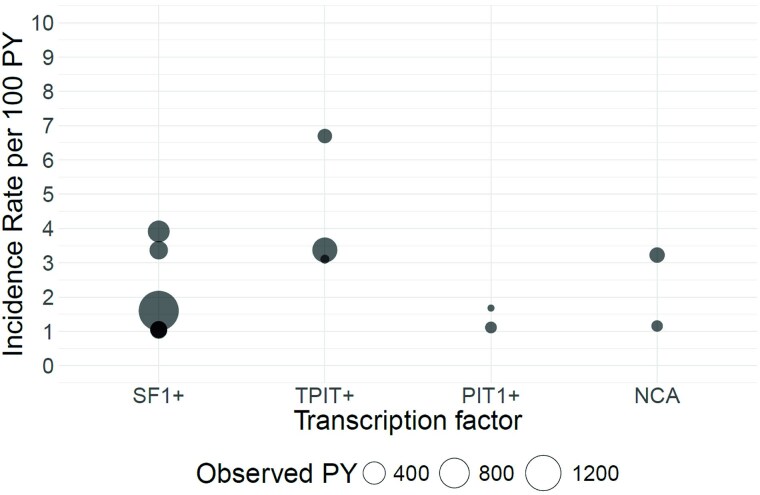
Bubble plot of incidence rates of postoperative radiotherapy per 100 patient-years (PYs) by transcription factor for comparison group 1. Every bubble represents the incidence rate calculated based on individual study data. Bubble size represents the observed patient years per subtype or per comparison. Comparison group 1: cross-comparisons between cell lineage-specific transcription factor-positive (SF1+, TPIT+, and PIT1+) NFPAs and cell lineage-specific transcription factor-negative (NCAs) NFPAs. Immunohistochemistry of adenohypophyseal hormones is not taken into account. Abbreviations: NCA, null cell adenoma; NFPAs, non-functioning pituitary adenomas; PIT1, pituitary transcription factor 1; PY, patient-year; SF1, steroidogenic factor 1; TPIT, T-box family member TBX19.

Postoperative radiotherapy was reported in 10 studies, while radiotherapy prior to surgery was described in a minority of patients in 3 studies (see Table 3 and Fig. 5). Reasons for ineligibility for meta-analysis were not meeting the inclusion criteria (49); the definition of reintervention, including surgery and/or radiotherapy (40); and selective reporting of the use of postoperative Gamma Knife treatment (only for patients who underwent treatment prior to progression of residual tumor) (6).

The mean pooled IRs of postoperative radiotherapy per 100 PYs could be calculated for SF1+ (IR 1.90; 95% CI, 0.88-3.28, 6 studies, 2730.4 observed PY, *I^2^* 51%, 95% PrI 0.88-3.28, low quality of evidence), and TPIT+ NFPAs (IR 3.94; 95% CI, 0.71-9.35, 3 studies, 702.8 observed PY, *I^2^* 26%, 95% PrI 0.0-41.34, moderate quality of evidence) only, both after first and/or repeat surgery (Table 7).

**Table 7.** Incidence rate of postoperative radiotherapy per 100 patient-years

Incidence rate of postoperative radiotherapy per 100 PYs after first and/or repeat surgery							Incidence rate of postoperative radiotherapy per 100 PYs after (probable) first surgery					
	No. of studies	n/N*^a^*	Incidence rate (95% CI)	*I^2^*, %	95% PrI	Quality of evidence (GRADE)	No. of studies	n/N*^a^*	Incidence rate (95% CI)	*I^2^*, %	95% PrI	Quality of evidence (GRADE)
**Comparison group 1**												
SF1+	6	53/2730.4	1.90 (0.88-3.28)	51	0.15-5.34	⊕⊕◯◯	2	NA	NA	NA	NA	NA
TPIT+	3	28/702.8	3.94 (0.71-9.35)	26	0.00-41.34	⊕⊕⊕◯	0	NA	NA	NA	NA	NA
PIT1+	2	NA	NA	NA	NA	NA	1	NA	NA	NA	NA	NA
NCA	2	NA	NA	NA	NA	NA	1	NA	NA	NA	NA	NA

Pooled incidence rates of postoperative radiotherapy per 100 PYs per histopathological subtype after first and/or repeat surgery and after first surgery. Comparison group 1: cross-comparisons between cell lineage-specific transcription factor–positive (SF1+, TPIT+, and PIT1+ NFPAs) and cell lineage-specific transcription factor–negative (NCAs) NFPAs. IHC of adenohypophyseal hormones is not taken into account. Calculation of incidence rates of postoperative radiotherapy per 100 PYs after first and/or repeat surgery and after first surgery was not possible for comparison groups 2 and 3 due to limited number of studies.

Abbreviations: IHC, immunohistochemistry; NA, not applicable (due to insufficient studies); NCA, null cell adenoma; NFPA, non-functioning pituitary adenoma; PIT1, pituitary transcription factor 1; PrI, prediction interval; PY, patient year; SF1, steroidogenic factor 1; TPIT, T-box family member TBX19. ⊕◯◯◯, very low quality of evidence; ⊕⊕◯◯, low quality of evidence; ⊕⊕⊕◯, moderate quality of evidence; ⊕⊕⊕⊕, high quality of evidence.

^a^The amount of patients with at least one postoperative radiotherapy treatment per the amount of observed PYs.

In total, 6 of 723 (0.8%) patients and a maximum of 4 of 228 (1.8%) patients were treated with prior radiation, respectively.

Meta-analyses of IRRs of postoperative radiotherapy per 100 PYs were not appropriate due to the limited number of studies. No statistically significant IRRs in individual studies were found when comparing the frequency of postoperative radiotherapy in NCAs with SF1+ NFPAs (IRR per 100 PY 6.13; 95% CI, 0.72-52.46 based on 5-year follow-up after first surgery (35), and 0.72; 95% CI, 0.10-5.34 based on 7.2 years of follow-up after first and/or repeat surgery (42)). Based on one cohort study, TPIT+ NFPAs were more frequently treated with postoperative radiotherapy compared with SF1+ NFPAs (IRR per 100 PY 2.11; 95% CI, 1.14-3.90 based on 1.2-year follow-up) (12), with a similar trend in the other cohort including both subtypes (IRR per 100 PY 1.99; 95% CI, 0.77-5.15 after 7.2 years of follow-up) (42). One study including all subtypes found the highest IRs per 100 PYs for TPIT+ (IR 3.37; 95% CI, 1.94-5.19), followed by SF1+ NFPAs (IR 1.60; 95% CI, 1.03-2.29), NCAs (IR 1.16; 95% CI, 0.00-4.97), and PIT1+ NFPAs (IR 1.11; 95% CI, 0.00-4.79), although CIs overlapped (42).

#### Comparison group 2

Due to the restricted number of studies, meta-analyses of IRs and IRRs of postoperative radiotherapy per 100 PYs could not be calculated for any of the subtypes nor comparisons (Supplementary Fig. S5 (19)).

#### Comparison group 3

Due to limited studies, meta-analyses of IRs and IRRs of postoperative radiotherapy per 100 PYs could not be performed for any of the subtypes and comparisons (Supplementary Fig. S6 (19)).

### Heterogeneity

More than half of the analyses of pooled estimates per histopathological subtype were affected by substantial to considerable heterogeneity, with wide 95% PrI. Possible explanations could be differences in study population, study design (eg, sample size, time of follow-up, and setting), and definitions of the outcomes measured. The analyses of the pooled estimate PRRs of CSI exhibited lower heterogeneity. An insufficient number of studies prevented us from conducting subgroup analyses other than the pooled estimates at time of the (probable) first surgery.

### Sensitivity Analyses

The observed effects and direction of effects did not change substantially across all possible sensitivity analyses (Supplementary Tables S3-S20 (19)). However, precision and statistical significance rapidly decreased due to the limited number of studies and small sample sizes.

### Publication Bias

Formal tests for asymmetry (Egger test) did not indicate publication bias for the subtype comparisons on CSI (Supplementary Fig. S7 (19)).

### Quality of Evidence

Overall, the quality of evidence was considered “low” or “very low” (Supplementary Tables S21-S23 (19)). Reasons for downgrading were serious risk of bias, between-study heterogeneity, small sample sizes, or the 95% CI containing 0.

## Discussion

This systematic review and meta-analysis represents the first to provide a comprehensive summary of the clinical behavior, measured as CSI, recurrence, and multimodality treatment of clinically NFPAs, categorized by cell lineages as defined by the WHO 2017/2022 classification. We included the PRRs for subtype comparisons, as well as the RRRs and IRRs per 100 PYs, which enabled us to compare cohorts with different follow-up times. Overall, our analyses show evidence of more aggressive clinical behavior of NCAs and TPIT+ NFPAs compared with SF1+ and PIT1+ NFPAs. CSI was more prevalent in NCAs and TPIT+ NFPAs compared with SF1+ (PRR 1.60 and 1.43, respectively), and NCAs compared with PIT1+ NFPAs (statistically significant difference compared with NCAs [PRR 1.44], a trend compared with TPIT+ NFPAs [PRR 1.51]). The RRs and the need for multimodality treatment, consisting only of postoperative radiotherapy, were higher in TPIT+ NFPAs than in SF1+ NFPAs (RR 3.64 per 100 PYs vs 2.05 per 100 PYs, and IR 3.94 per 100 PYs vs 1.90 per 100 PYs, respectively). However, studies that included both subtypes were scarce and meta-analyses of RRRs and IRRs were not feasible.

Due to the limited number of studies, we could not properly assess the differences in CSI, recurrence, and the use of postoperative radiotherapy in comparison groups 2 and 3. The histopathological subtypes in comparison group 2 all demonstrate negative IHC results for adenohypophyseal hormones. The effect of the addition of the cell lineage-specific TFs is mainly observed in comparison groups 2 and 3, as most of them can now be assigned to 1 of the 3 cell lineages and are now part of those subtypes in comparison group 1.

In contrast with other studies and reviews, we compared all histopathological subtypes separately rather than comparing one histopathological subtype with all other subtypes of NFPAs combined. This approach allowed for a more precise analysis of the risk of invasion, recurrence, and multimodality treatment among distinct histopathological subtypes of clinically relevant NFPAs than would have been possible in single studies. Previous systematic reviews that assessed the clinical behavior and natural history of NFPAs applied the WHO 2004 histopathological classification, and did not cross-compare all histopathological subtypes (10, 57, 58). A previous systematic review by Fountas et al (2019) (10) reported recurrence rates of 5.41 and 4.88 per 100 PYs for SCAs at less than 5 and more than 5 years of follow-up, and concluded that SCAs do not have higher RRs compared with other NFPAs. The RR for TPIT+ NFPAs found in this study is lower (3.64 per 100 PYs) than that reported by Fountas et al (2019) (10), and we did find higher RRs compared with SF1+ NFPAs. These discrepancies in outcomes can be explained by the use of different definitions: Fountas et al (10) defined SCAs as IHC ACTH+ only, thereby omitting the ACTH-negative TPIT-positive (TPIT+/ACTH−) NFPAs. Moreover, the same study compared SCAs to a mixture of other NFPAs rather than comparing them to specific subtypes, which may also affect the outcomes given the distinct clinical behavior of NFPA subtypes identified by our systematic review.

Another strength of the present review is that we performed sensitivity analyses of cohorts that excluded patients with prior pituitary surgery. This was done to rule out potential selection bias in the primary outcomes resulting from patients in whom recurrent surgery was indicated (59). Although the precision of the estimates in these sensitivity analyses declined due to the limited number of included studies, the direction of the effects was consistent. Furthermore, we conducted sensitivity analyses based on the different definitions of CSI to ensure the robustness of our findings.

The studies identified by our systematic review have several limitations. First, the accuracy of the estimates may have been affected by the quality and risk of bias of the individual studies. More than half of the studies had a moderate to high risk of bias. The main reasons for ascribing a moderate or high risk of bias were the lack of a clear definition and description of the measurement of prognostic factors and outcomes. In addition, almost none of the studies corrected for confounding. Second, the length of follow-up differed across studies, which represents another considerable limitation. Despite standardizing the RRs and IRs of multimodality treatment per 100 PYs, subgroup analysis of studies with relatively short and long follow-up durations was not possible due to the limited number of studies. Selection bias due to loss to follow-up may have influenced the results, as attrition was not always reported. Third, the lack of reporting of the antibodies used for IHC of TFs and adenohypophyseal hormones, along with the cutoff value at which positivity was established, and the variations in quality of the different antibodies, could have introduced variability and potential bias into our analyses of histopathological subtypes. Coexpression of multiple TFs has been reported by IHC (60, 61) and by methylome and/or transcriptome analysis (39, 62) in NFPAs. However, the prevalence and clinical effect of coexpression remains unclear. As our focus was on discriminating between TFs, we have excluded cases of coexpression where possible. However, it cannot be ruled out with certainty that undetected coexpression may have occurred in these studies. Finally, the absence of individual patient data in the majority of studies precluded the conducting of more detailed analyses, such as the exploration of heterogeneity and the control of potential bias.

In conclusion, the use of cell lineage-specific TFs by IHC enables the detection of histopathological subtypes of NFPAs with distinct clinical behavior. TPIT+ NFPAs and NCAs show a more aggressive behavior in terms of CSI compared with SF1+ and PIT1+ NFPAs. It appears that TPIT+ NFPAs may exhibit higher RRs and a more frequent need for postoperative radiotherapy than SF1+ NFPAs. However, this could not be substantiated by direct comparison due to the limited number of studies available.

The use of IHC of solely adenohypophyseal hormones significantly underestimates the prevalence of SCAs. At the same time, TPIT+/H− NFPAs appear to contribute equally to the more aggressive clinical behavior of SCAs in comparison to other NFPAs subtypes as TPIT+/H+ NFPAs do. The currently available clinical diagnostics indicate that only a small proportion of PAs are true NCAs. Despite their reduced prevalence, these NCAs exhibit an aggressive clinical potency. We therefore support the statement that combined clinical, biochemical, and IHC analyses of TFs and adenohypophyseal hormones provide a sliding scale that encompasses not only the distinction between clinically silent, whispering, and functioning adenomas, but also the differentiation between true NCAs (TF−/H− NFPA) and silent PAs (TF+/H± NFPA) (63). The observed differences in clinical behavior may be attributed to the abnormal transcriptomic changes that follow somatic mutations. These changes can result in the upregulation or downregulation of oncogenic or tumor suppressor genes, as well as affect cell proliferation and epithelial development. Somatic mutations in *USP8*, which are specific to the TPIT+ lineage, may serve as an illustrative example. However, it is important to note that cell lineage-specific mutations have been described for all cell lineages (64). The use of IHC of the cell lineage-specific TFs SF1, TPIT, and PIT1 enhances the diagnostic accuracy of clinically NFPA subtypes, enabling us to adjust clinical and radiological follow-up, and potentially treatment. Future research with clear definitions of CSI, recurrence, and multimodality treatment within large prospective cohorts with a histopathological diagnosis according to the WHO 2017/2022 is needed to further study the clinical behavior of the cell lineage-specific NFPA subtypes, and ultimately refine multidisciplinary clinical decision-making.

## Data Availability

Some or all data sets generated during and/or analyzed during the present study are not publicly available but are available from the corresponding author on reasonable request.

## Clinical Trial Information

PROSPERO registration number CRD42023435409 (registered 25-06-2023).

## Abbreviations

ACTH: adrenocorticotropin
CSI: cavernous sinus invasion
GTR: gross total resection
H: adenohypophyseal hormone
IHC: immunohistochemistry
IQR: interquartile range
IR: incidence rate
IRR: incidence rate ratio
MRI: magnetic resonance imaging
NCA: null cell adenoma
NFPA: non-functioning pituitary adenoma
PA: pituitary adenoma
PIT1: pituitary transcription factor 1
PitNET: pituitary neuroendocrine tumor
PR: prevalence rate
PrI: prediction interval
PRR: prevalence rate ratio
PY: patient-year
QUIPS: Quality In Prognostic Studies
RR: recurrence rate
RRR: recurrence rate ratio
SCA: silent corticotroph adenoma
SF1: steroidogenic factor 1
SGA: silent gonadotroph adenoma
TF: transcription factor
TPIT: T-box family member TBX19
WHO: World Health Organization
